# Mitochondrial Reactive Oxygen Species Production in Vascular Dementia Following Experimental Diabetes

**DOI:** 10.3390/cells14161260

**Published:** 2025-08-15

**Authors:** Ed Wilson Santos, Subika Khatoon, Yun-Min Zheng, Yong-Xiao Wang

**Affiliations:** Department of Molecular and Cellular Physiology, Albany Medical College, 47 New Scotland Avenue, Albany, NY 12208-3479, USA; cavalce@amc.edu (E.W.S.); khatoos@amc.edu (S.K.)

**Keywords:** cerebral artery smooth muscle cell, cognitive impairment, dementia, diabetes, mitochondria, oxidative stress

## Abstract

Type 1 diabetes (T1D) is a serious disease which affects millions of people worldwide and is a major factor for vascular contributions to cognitive impairment and dementia (VCID). In this study, we first characterized cognitive and memory impairments, then evaluated their underlying molecular mechanisms, and finally determined sex-dependent effects in male and female mice with streptozotocin (STZ)-induced T1D. Our findings indicated that significant cognitive impairment, memory loss, and vascular dementia occurred in male and female T1D mice. Cerebral artery (CA) blood flow was greatly reduced in the various brain regions tested. ROS generation in isolated cells, mitochondria, and mitochondrial complex III from CA smooth muscle cells (CASMCs) were all increased in T1D. DNA damage and Tau phosphorylation in CASMCs were largely increased. Linear regression analysis revealed that T1D-induced increased blood glucose was highly correlated with increased ROS production and increased VCID. Taken together, we conclude that T1D causes increased mitochondrial complex III ROS production, DNA damage, and Chk2 phosphorylation in CASMC, thereby leading to vascular dementia in both male and female mice; our results further demonstrate that mitochondrial complex III ROS-mediated DNA damage is more significant in male than female mice, which contributes to more serious vascular dementia in the former than the latter.

## 1. Introduction

Diabetes refers to a group of common and serious diseases. Currently, more than 537 million people have diabetes across the world, and it is predicted that diabetes cases will increase to 643 million by 2030 and 783 million by 2045; furthermore, over 38.4 million people have diabetes in the United States [1,2]. There are two major forms of diabetes, type 1 and type 2 (T1D and T2D). T1D can be brought on by a variety of pathogenic mechanisms to result in insulin insufficiency [3].

Patients with T1D are more prone to having nervous system pathologies. Higher blood glucose levels in T1D may abnormalize cerebral artery blood flow (CABF), which changes brain transport, brain metabolism, and histological damage, leading to vascular contributions to cognitive impairment and dementia (VCID) as well as other neural diseases [4]. Mitochondria have essential functions in vascular smooth muscle cells (SMCs), particularly by producing energy in the form of ATP to sustain normal cell activities [5]. Mitochondria have also been known to act as a primary site to produce reactive oxygen species (ROS) in vascular SMCs. As an important signaling molecule, ROS mediates pulmonary vasoconstriction and vasoremodeling, leading to the development and progression of pulmonary hypertension [5,6]. Mitochondrial ROS may also affect CA functions contributing to brain damage in stroke patients [7].

Sex differences have a major impact on diabetes generation, progression, pathophysiology, vascular complications, and outcomes [8]. Diabetic vascular neuropathy is seen more often in men than in women [9]. In support, anaerobic glycolysis in various brain regions is significantly altered in male mice, but not in female mice [9]. Sex plays an important role in determining the occurrence and progression of neuropathies [10]. Indeed, sex-dependent metabolic changes in the brain have been observed in T1D mice [9]. Moreover, cognitive decline is found to be a serious neurological complication in T1D due to oxidative stress [11].

In this study, our first goal was to characterize VCID features in male and female T1D mice. As the second goal, we sought to elucidate the molecular mechanisms underlying T1D-induced VCID. Finally, we wanted to determine potential sex differences in T1D-evoked VCID in male and female mice. The findings obtained from this research may not only have a significant impact on enhancing our current knowledge about the development of VCID in T1D, the underlying mechanistic signaling process, and sex differences in mediating T1D-induced VCID in males and females, but also help to create novel and specific therapeutic targets for VCID, diabetes, and other relevant diseases.

## 2. Materials and Methods

### 2.1. Generation of T1D

All experiments using animals were conducted in compliance with the National Institutes of Health guidelines and approved by the Institutional Animal Care and Use Committee (IACUC) at Albany Medical College. Animal research reporting of in vivo experiments (ARRIVE) guidelines were followed to prepare this manuscript. We used 10 ± 3-week-old male and female C57BL/6J mice from Jackson Laboratories (Bar Harbor, ME, USA). The mice were randomly assigned to caged housed groups (4–5 per cage) at 70–72 °F, 30–70% humidity, with a 12 h light/dark cycle. The mice were subjected to an intraperitoneal single injection of 180 mg/kg of streptozotocin (STZ) to induce T1D, as we reported previously [12]. Blood glucose was measured using a OneTouch^TM^ Glucose Meter (LifeScan Europe GmbH, Zug, Switzerland) through a small cut at the end of the tail. Animals exceeding 280 mg/dL in blood glucose levels were considered diabetic [12].

### 2.2. Voluntary Urination Assay

The mice were individually housed with filter paper for 1 h to establish voluntary urination. The diameters of the urine spots were quantified per mouse and averaged.

### 2.3. Measurement of Visceral Fats

Following euthanasia, the retroperitoneal adipose pads were collected and immediately weighed.

### 2.4. Behavior Tests

#### 2.4.1. Acclimatization and Open Field

The mice were placed in a room where behavioral tests were performed 1 h before the beginning of acclimation. After acclimation, the mice were then placed in the arena (50 cm × 50 cm), with one mouse per arena. The mice were placed at identical points in each arena. The mice were then given 10 min to explore the open field. The mice were returned to their post-test cages after 10 min, and the arena was cleaned between each set of animals. As a measure of anxiety-like behavior, the total time of the experiment is quantified for time spent in the corners of the arena vs. time spent in the middle region [13].

#### 2.4.2. Object Position Tests

The mice were placed in an arena with two identical objects. The objects were set in the same exact position in each arena. The mice were then given 10 min to inspect the indistinguishable objects. The mice were returned to their post-test cages after 10 min, and the arena and objects were cleaned between each set of mice. The mice returned to their cages for a duration of 20 min. The position of one identical object was then changed, and the mice were reintroduced into the arena. A total of ten minutes was allotted for object exploration [13]. The object position and novel object’s discrimination index (DI) were computed [14], in which DI = (time spent with a new object (or location) − time spent with a familiar object (or location))/(time spent with a new object (or location) + time spent with a familiar object (or location)). A positive score suggests that more time is spent researching the novel object (or location). A discrimination index of 0 indicates that both objects were investigated equally (or location). Using automated tracking software, the open field, object position, and NORT were video recorded and evaluated (ANY-maze 5.1, Stoelting, Wood Dale, IL, USA).

#### 2.4.3. Novel Object Recognition Test (NORT)

The mice were placed in an arena containing one familiar (from the previous phase) and one novel object. For each arena, the novel object was placed in the same area. The mice were then permitted to explore the arena with the objects for 10 min [13]. The mice were then returned to their post-test cages, and the arena and objects were cleaned between groups of mice.

The novel object discrimination index (DI) was computed as described above. If mice did not reach a minimum of 20 s of exploration for both objects in 10 min, we excluded them from analysis, as it could not be confirmed that they spent enough time exploring to discriminate [15].

#### 2.4.4. Nest Building Test

Nest building is an innate behavior in male and female rodents, even when raised in laboratory settings, as a gauge of their overall well-being and as an ancillary assessment to predict possible decline in cognition. The mice were housed individually in chip litter with 2 squares of cotton material for 16–18 h. After this time, the mice were cautiously removed from the cages and housed in batches in their home cages. Nests were graded by three independent raters blinded to each condition. The nests were graded on a scale of 5, with 1 being the lowest possible rating and 5 being the highest. The results were based on the rigorousness with which the mice tore up the nesting material, forming a compact crater-shaped nest confined to a corner of their cage [16].

### 2.5. Cerebral Blood Flow

Images of the cortical surface vasculature were obtained using a laser speckle contrast imaging device. The animals were administered avertin anesthesia prior to imaging and skull exposure through a ~2 cm vertical incision in the skin and connective tissue. Images were acquired for 10 min at a rate of 10 s/image utilizing a full-field laser speckle imager (FLP1, Moor Instruments, Wilmington, DE, USA). The images were analyzed using FLP1 Review V4.0 software (Moor Instruments). On each image, the cerebral fields of interest were drawn in the left and right frontal, parietal, temporal, and pial zone anastomotic areas. Mean CBF values were obtained for each region of interest (ROI) [13].

### 2.6. Preparation of Cerebral Artery Smooth Muscle Cells (CASMCs)

Freshly isolated CASMCs were obtained using a similar procedure as reported previously [12]. Briefly, mice were anesthetized with an intraperitoneal injection of avertin (250 mg/kg) after 60 days of STZ injection. Then cerebral arteries were digested using a two-step enzymatic method. Enzymes were dissolved in low Ca^2+^ (100 µM) PSS containing (mg/mL) 1.5 papain, 0.4 dithioerythritol, and 1.0 bovine serum albumin (BSA) for 10 min, followed by 1.0 collagenase II, 1.0 collagenase F, 1.0 dithiothreitol, and 1.0 BSA for 10 min. The digested CAs were gently triturated to harvest SMCs.

### 2.7. Isolation of Mitochondria

Mitochondria were isolated from the CASMCs by differential centrifugation. All steps below were performed at 4 °C. Isolated CAs were placed in an all-glass Dounce homogenizer containing 1 mL of isolation buffer consisting of (mM) 215 mannitol, 75 sucrose, 20 4-(2-hydroxyethyl)-1-piperazineethanesulfonic acid (HEPES), 1 ethylene glycol tetra acetic acid (EGTA), and 0.1% bovine serum albumin (BSA, pH 7.2), and homogenized. The homogenate was spun twice at 1300× *g* in an Eppendorf microcentrifuge for 5 min. The resulting supernatant was subsequently centrifuged at 13,000× *g* for 15 min. The pellet was resuspended in 200 µL PBS [17].

### 2.8. ROS Generation Assays

Here, we first used lucigenin, perhaps the most commonly used chemiluminescent probe, as we reported previously [18], to primarily detect superoxide anion (O_2_^−^) generation in CASMCs. Mice were anesthetized with an intraperitoneal injection of avertin (250 mg/kg) at 60 days after STZ injection. CASMCs was isolated in physiological saline solution (PSS), and lysates (50 μg protein samples) were incubated with lucigenin (20 μM, Abcam^TM^, Whaltam, MA, USA). After 10 min, the emitted chemiluminescence derived from lucigenin was detected at 455 nm using a FlexStation-III microspectrophotometer (Molecular Devices^TM^, San Jose, CA, USA). Next, we used this chemiluminescent probe to determine O^−^ generation in isolated mitochondria (50 μg protein) from freshly isolated CASMCs. CASMCs were isolated using a two-step enzymatic method as we reported previously [12,19]. CAs were digested in low-Ca^2+^ (100 µM) PSS containing (mg/mL) 1.5 papain for 10 min, followed by 1.0 collagenase II and 1.0 collagenase F for 10 min, as we reported previously [12,18,20]. The isolated SMCs were also confirmed using immunofluorescent staining [18,20] subjected to differential centrifugation to obtain mitochondria [19,21]. Isolated mitochondria (50 μg protein) were added in microplate wells containing respiration buffer that included 5 mM pyruvate, 2.5 mM malate, and lucigenin 20 μM. After incubation for 10 min, the emitted chemiluminescence was measured at 455 nm.

We further detected O^−^ generation in isolated mitochondrial complex III obtained from isolated CASMCs. To isolate mitochondrial complex III, a specific immunocapture kit (Abcam^TM^, Whaltam, MA, USA) was utilized according to the manufacturer’s instructions, as previously illustrated [21]. To evaluate lucigenin in complex III, the same protocol was used; just changing the respiratory buffer to 40 μM reduced decylubiquinone, 2 mM potassium cyanide, 50 μM cytochrome c, and lucigenin 20 μM [21]. The initial (maximum) values of lucigenin-derived chemiluminescence were normalized to the control group.

The *N*-acetyl-3,7-dihydroxyphenoxazine (Amplex UltraRed, Invitrogen^TM^, Carlsbad, CA, USA) assay, developed by Molecular Probes, is based on the horseradish peroxidase-catalyzed oxidation of the nonfluorescent molecule Amplex. Fifty micrograms of protein from isolated CASMCs, mitochondria, or complex III were incubated with Amplex (50 μM) and HRP (5 μM). After incubation for 10 min, fluorescence was measured at 37 °C using the FlexStation-III spectrophotometer with an excitation wavelength of 530 nm and emission wavelength of 590 nm [19,22].

Total ROS production was measured with dichlorodihydrofluorescein diacetate (DCF). CASMCs or isolated mitochondria (50 μg protein) were added in microplate wells containing respiration buffer that included 5 mM pyruvate, 2.5 mM malate, 10 μM dichlorodihydrofluorescein diacetate (H_2_DCF/DA, Sigma-Aldrich^TM^, St. Louis, MO, USA) and 5 μM horseradish peroxidase (HRP). After incubation for 10 min, the fluorescence was measured at 37 °C using the FlexStation-III spectrophotometer with an excitation wavelength of 485 nm and an emission wavelength of 532 nm. ROS production was determined by subtracting the fluorescence intensity measured in control wells that contained assay buffer without mitochondria. The lucigenin chemiluminescence probe was utilized (mainly to quantify O^2−^) as a complementary approach to study [ROS]_i_ in CASMCs. To evaluate DCF in complex III, the same protocol was used; just changing the respiratory buffer to 40 μM reduced decylubiquinone, 2 mM potassium cyanide, and 50 μM cytochrome c.

We injected Mito-Tempo (Sigma-Aldrich^TM^, St. Louis, MO, USA) for five consecutive days (1 mg/Kg/day) to evaluate the antioxidant effects in diabetics. This chemical is a mitochondrial-targeted antioxidant that acts as a superoxide dismutase mimetic, meaning it mimics the action of the enzyme superoxide dismutase to scavenge superoxide radicals.

### 2.9. DNA Damage

Chk2 is a key component of the DNA damage response (DDR) pathway, which regulates how cells respond to DNA double-strand breaks (DSBs). DSBs can occur due to normal metabolism, exposure to toxins, or other factors, and can lead to chromosomal rearrangements or loss of genetic material. DNA damage and an inadequate DDR are associated with the development of neurodegenerative diseases. Chk2 can enhance tau toxicity, which is the abnormal metabolism of tau protein detected in the brains of people with neurodegenerative diseases. Chk2 phosphorylation is a standard marker for DNA damage [23]; therefore, its expression level was assessed using Western blotting with Chk2 antibody (1:1000, Abcam^TM^, Whaltam, MA, USA).

### 2.10. Tau Protein Phosphorylation

Western blotting of phospho-tau-S262 and total tau in CA were performed with anti-p-tau-S262 anti-total-tau antibody (1:1000, Abcam^TM^, Whaltam, MA, USA). Quantification of phospho-tau-S262 over the total amount was calculated. We focused on phospho-tau-S262, since their phosphorylation was known [24].

### 2.11. Statistical Analysis

The data are presented as the mean ± standard error of the mean with a 95% CI (confidence interval). Statistical comparations between groups were performed with two-way ANOVA followed by Tukey’s multiple comparisons test using GraphPad Prism 10.5.0. Differences at *p* < 0.05 were considered statistically significant.

## 3. Results

### 3.1. Sex-Specific Changes in Body Weight, Glucose, Visceral Fat, and Urination in T1D Mice

**Figure 1:**
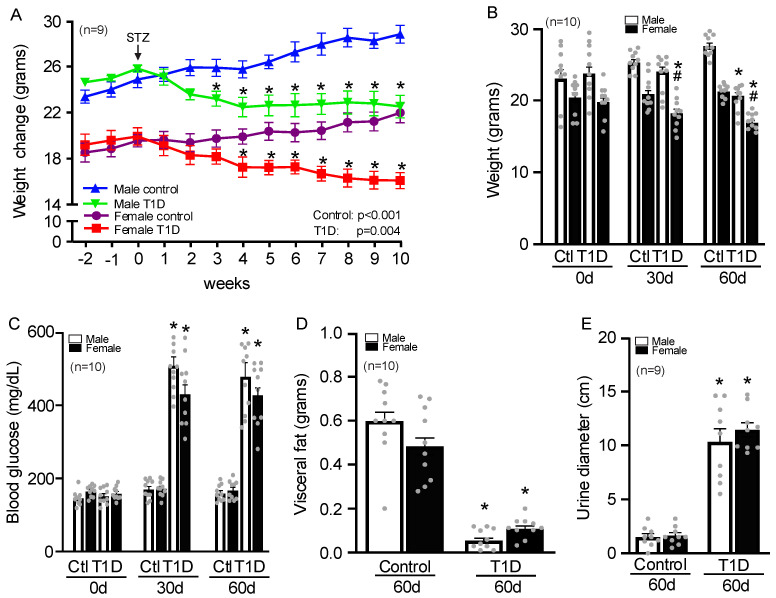
T1D mice showed a significant decrease in weight and visceral fat; however, they showed an increase in blood glucose levels and voluntary urination. STZ injection caused a decrease in body weight in both sexes (**A**,**B**) with a significant increase in blood glucose levels (**C**), and a decrease in visceral fat (**D**). Voluntary urination was higher in T1D after 60 days (**E**). The diameters of urinary spots are measured and averaged per mouse. The data are presented as the mean with a 95% CI (n = 9–10/group), analyzed by two-way ANOVA. * *p* < 0.05 compared with control; ^#^ *p* < 0.05 compared with female. T1D = Type 1 diabetes; 30d = 30 days; 60d = 60 days.

Male mice (Figure 1A) had a higher average weight than female mice at the start of the trial. Both showed statistically significant weight loss after 30 days of STZ injection, which continued until 60 days (Figure 1B). After 60 days, males reached levels close to 500 mg/dL, while females reached levels close to 400 mg/dL (Figure 1C). Males and females showed similar visceral fat declines at the time of euthanasia (Figure 1D). Voluntary urination increased in both sexes and remained high throughout the research, with spots with diameters close to 11 cm (Figure 1E).

### 3.2. Sex-Specific Changes in Behavior Tests

**Figure 2:**
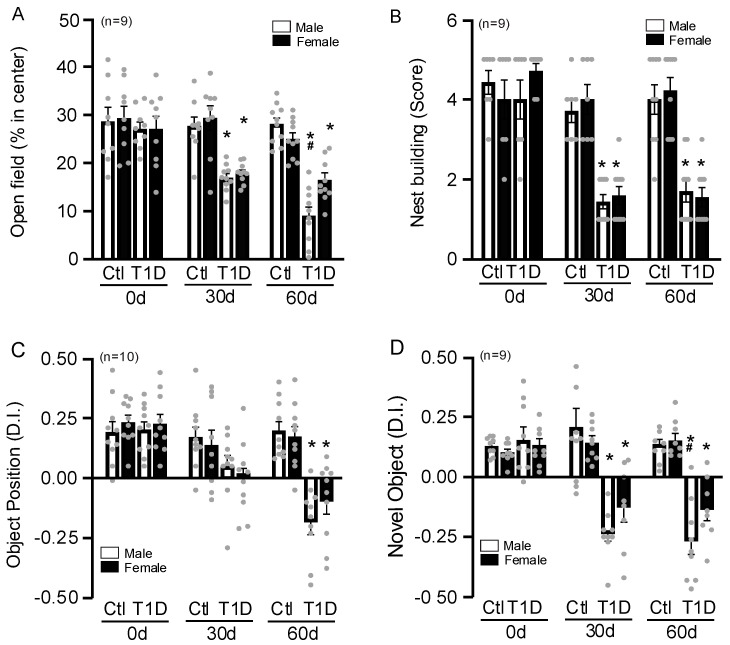
Behavior tests show that T1D can lead to behavioral changes, such as increased anxiety, decreased cognition, spatial memory, and daily tasks. Open field test (**A**). Nest building (**B**). Nests are then scored based on completeness. Object position test (**C**). The total investigation time of the novel location of the object is then calculated as a percentage of the time exploration time of the 2 objects (1 familiar and 1 novel location). Novel object recognition test (**D**). The total investigation time of the novel object is then calculated as a percentage of the exploration time of the 2 objects (1 familiar and 1 novel). Data was obtained from five separate experiments. Each dot represents one mouse. Analyzed by two-way ANOVA. * *p* < 0.05 compared with the control; ^#^ *p* < 0.05 compared with males. Data are presented as mean with 95% CI (N = 9–10/group). T1D = Type 1 diabetes; 30d = 30 days; 60d = 60 days.

Anxiety-like behavior in mice can be measured using the open field test. Less anxious mice would spend more time exploring the central area of the test box. Both male and female mice with T1D at 60 days had a more significant reduction in time in the center of the box, showing the cumulative and long-term effect. It is important to note that males had significantly less time at the center compared to females, demonstrating a more pronounced anxiety behavior than the other groups (Figure 2A). Nest building tests can be used to show the daily routine of mice. In this study, we discovered that diabetic mice performed worse than control mice at 30 and 60 days similarly in both sexes (Figure 2B). The object position test demonstrates alterations in cognition and memory. After 4 weeks, this test did not reveal any appreciable differences between the sexes. However, after 60 days, there was a significant decline in recognition in both groups (Figure 2C). Normal, healthy mice exhibited a preference for a new object over a familiar one, but animals with cognitive impairment or abnormalities spent the same amount of time studying both types of objects. Indices like the discrimination index are used to highlight this preference. Biologically, a positive DI reflects preserved discrimination ability, indicating that memory, sensory perception, and motivation remain intact to differentiate and preferentially explore the target stimulus. In an object recognition test, a positive DI suggests that the animal recognizes the familiar object and allocates more time to exploring the novel one. Researchers can identify preference among unfamiliar things in behavioral experiments using discrimination indices. According to tests examining the novel object recognition tests (NORTs), control mice tended to inspect the novel object (as demonstrated by a positive discrimination index value), but STZ-injected animals displayed a reduced ability to recall the objects (Figure 2D). We found that males showed an even bigger decline in novel object recognition at 60 days. Therefore, these results may suggest a higher decline in behavioral functions in males.

### 3.3. STZ-Injected Animals Have Decreased Cerebral Blood Flow

**Figure 3:**
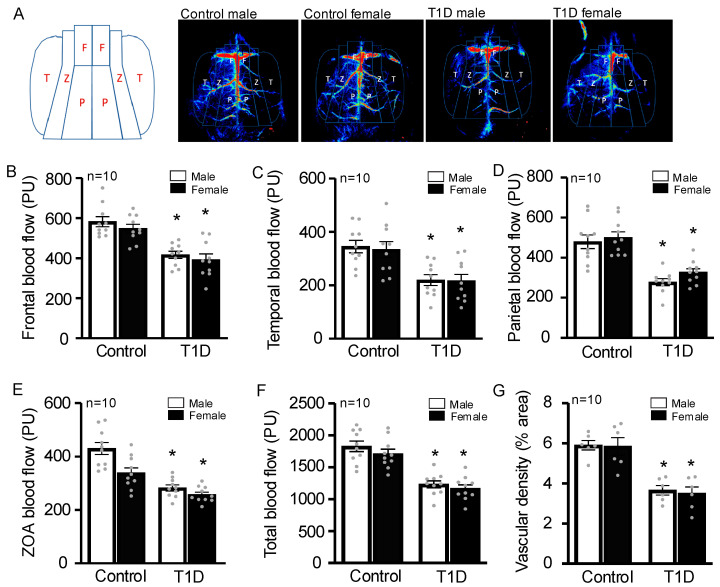
CBF is decreased in all STZ-injected mice. CBF was measured after 60 days via laser speckle contrast imaging in T1D mice after 60 days of STZ injection. Regions of interest were drawn for the frontal [F], parietal [P], ZOA (zone of anastomoses) [Z], and temporal [T] cortical regions. Representative images are shown (**A**). Results show that frontal (**B**), temporal (**C**), parietal (**D**), ZOA (**E**), and total (**F**) regions and vascular density (**G**) had a significant decrease in blood flow in all STZ-injected groups after 60 days of injection. Analyzed by two-way ANOVA. * *p* < 0.05 compared with control. Data are presented as mean with 95% CI (n = 10/group). PU = perfusion units. T1D = Type 1 diabetes.

Decreased CBF has been reported in cases of diabetes, and there are studies linking hypoperfusion to impaired cognitive function [25]. CBF was measured using a full-field laser speckle imager (FLP1, Moor Instruments, Wilmington, DE, USA), and we observed that all animals injected with STZ demonstrated a reduction in CBF. There were no statistically significant differences between males and females. We designed four regions of interest (ROIs) (Figure 3A): frontal (Figure 3B), temporal (Figure 3C) parietal (Figure 3D), zone of pial anastomoses (ZOA) (Figure 3E), and total (Figure 3F). In all four ROIs, there was a significant reduction in all STZ-injected groups. We also measured the vascular density, and both sexes had a decrease (Figure 3G). CBF in T1D was greatly reduced, which could be responsible for the development of VCID.

### 3.4. Sex-Specific Changes in ROS Generation

T1D has been linked to an increase in ROS generation and/or a decrease in antioxidant defenses. Hyperglycemia stimulates ROS production via mitochondrial respiratory chain enzymes, NOS, xanthine oxidases, lipoxygenases, and peroxidases [19]. However, little is known regarding sex differences in ROS production in T1D.

**Figure 4:**
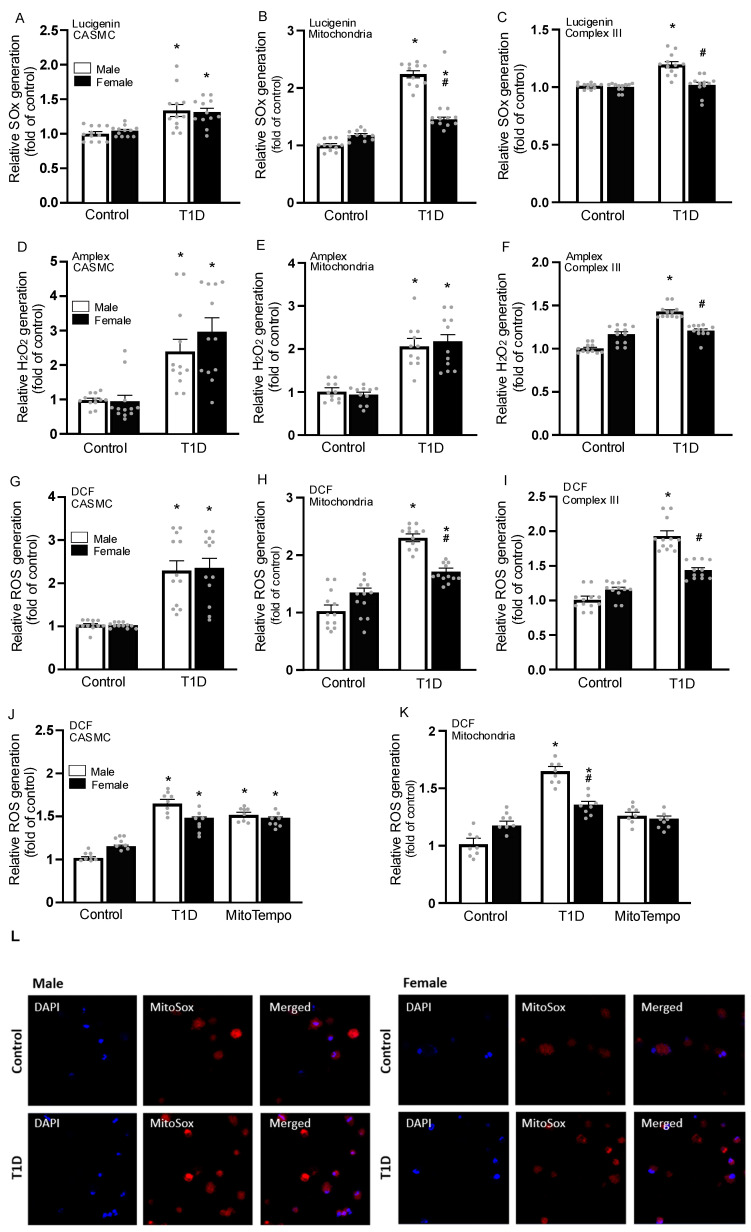
T1D increases ROS production in isolated CASMCs, mitochondria, and mitochondrial complex III. After 60 days, superoxide anion (O_2_^−^) production was determined by using Lucigenin chemiluminescence in CASMCs (**A**), isolated mitochondria (**B**), and isolated complex III (**C**). H_2_O_2_ generation was determined using Amplex UltraRed fluorescence in CASMCs (**D**), isolated mitochondria (**E**), and isolated complex III (**F**). Total ROS generation was determined using H_2_DCF/DA (DCF) fluorescence in the CASMCs (**G**), isolated mitochondria (**H**), and isolated complex III (**I**). DCF also will evaluated in CASMCs (**J**) and isolated mitochondria (**K**) from mice treated with the antioxidant Mito-Tempo for 5 days before euthanized. Representative images from CASMCs stained with MitoSox and DAPI (**L**). Data were obtained from three separate experiments. Each dot represents one mouse. Analyzed by two-way ANOVA. * *p* < 0.05 compared with control; ^#^ *p* < 0.05 compared with males. Data are presented as mean with 95% CI (n = 12/group). H_2_O_2_ = hydrogen peroxide; T1D = Type 1 diabetes. CASMC = cerebral artery smooth muscle cell.

We investigated superoxide anion production using Lucigenin and observed similar increases in T1D production in both sexes in CASMC (Figure 4A). In isolated mitochondria, T1D males had more production than females (Figure 4B). When examining mitochondrial complex III, we found that, like DCF and Amplex, only T1D males showed an increase in ROS (Figure 4C). Amplex UltraRed measurements of H_2_O_2_ production in isolated CASMCs, and isolated mitochondria, showed higher levels in both female and male T1D mice (Figure 4D,E); moreover, the increased ROS in isolated mitochondrial complex III were only shown in male, but not female, diabetic mice (Figure 4F). Using DCF, we measured total ROS production in CASMCs and found a similar increase in both female and male diabetic CASMCs (Figure 4G). When measured in isolated mitochondria (Figure 4H) and mitochondrial complex III (Figure 4I), T1D males had a greater increase in ROS production.

Furthermore, we aimed to evaluate ROS production following inhibition with Mito-Tempo. Total ROS levels were measured in CASMCs (Figure 4J), and isolated mitochondria (Figure 4K), with inhibition being more pronounced in the isolated mitochondrial fraction. Additionally, cells were stained with MitoSOX (Figure 4L) to assess differences in superoxide anion levels. Consistent with previous findings, diabetic cells exhibited a marked increase in ROS production.

### 3.5. Correlation Between Blood Glucose Levels and Behavioral Assessments

**Figure 5:**
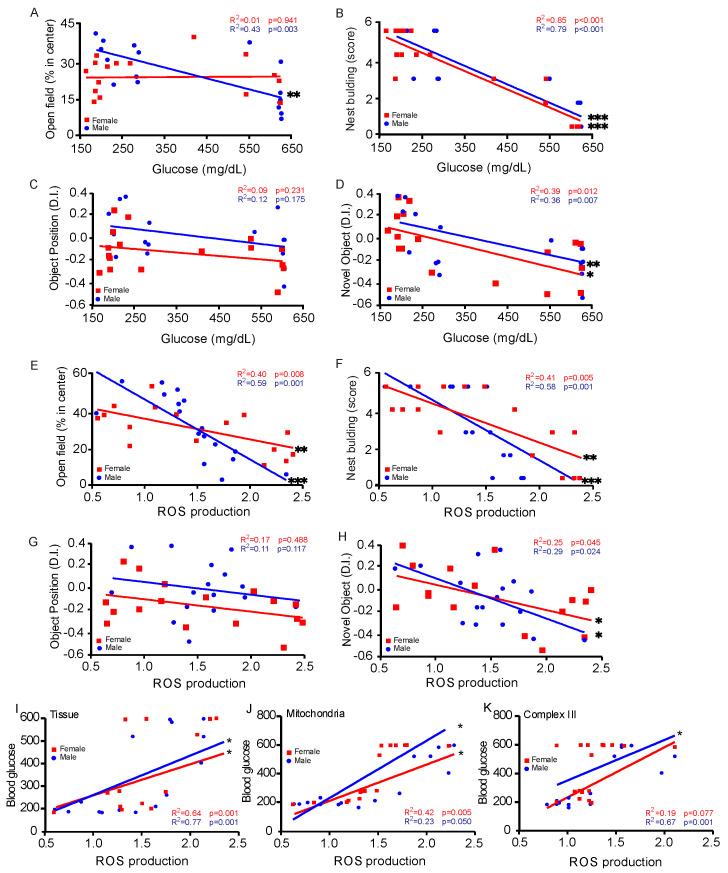
Correlations between behavior tests and blood glucose levels (**A**–**D**) and between ROS production (**E**–**H**) after 60 days show that T1D can lead to behavioral changes, such as increased anxiety, decreased cognition, spatial memory, and changes in daily tasks. Open field test (**A**,**E**): Total investigation time of the center region is calculated as a percentage of time in the center region. Nest building (**B**,**F**): Nests are scored based on completeness. Object position test (**C**,**G**): Investigation time of the novel location of the object is then calculated as a percentage of the exploration time of the 2 objects (1 familiar and 1 novel location). Novel object recognition test (**D**,**H**): Total investigation time of the novel object is then calculated as a percentage of the exploration time of the 2 objects (1 familiar and 1 novel). Positive correlation between blood glucose levels and ROS production in whole cells (**I**), isolated mitochondria (**J**), and isolated complex III (**K**). Data was obtained from five separate experiments. Each symbol represents one mouse. * *p* < 0.05; ** *p* < 0.01; *** *p* < 0.001. Data are presented as the mean with 95% CI (n = 16/group). T1D = Type 1 diabetes.

To determine how high blood glucose levels affected brain cells, we compared blood glucose levels with behavioral assessments. The results of the open field test revealed that there is a negative link between hyperglycemia in male but not in female mice; the latter spend less time exploring the center of the arena and exhibit greater anxiety as blood glucose levels rise (Figure 5A). The most significant negative connection was seen in both sexes during the nest construction test, where it was highest (Figure 5B). The object location test did not reveal any meaningful connections (Figure 5C). Between both sexes, male performance was greater in the NORT, which exhibited a negative connection (Figure 5D). We also correlated behavior tests with ROS production, and the data shows that there is a negative link between hyperglycemia that is more significant in males in open field (Figure 5E) nest building (Figure 5F) tests. Interestingly, no significant differences were detected in the object position test (Figure 5G). This finding may indicate that diabetes did not impair hippocampal-dependent spatial memory in this model. Alternatively, the four-week period may have been insufficient for the emergence of measurable deficits, or compensatory mechanisms—such as neural plasticity, recruitment of alternative brain regions, or neurochemical adaptations—may have preserved performance. NORT (Figure 5H) showed a similar decrease in both sexes.

To better understand the increased ROS production in T1D, we correlated blood glucose level and ROS production in CASMCs (Figure 5I), isolated mitochondria (Figure 5J), and isolated mitochondrial complex III (Figure 5K), where we were able to observe an extremely positive correlation in all cases.

### 3.6. DNA Damage in CASMC

**Figure 6:**
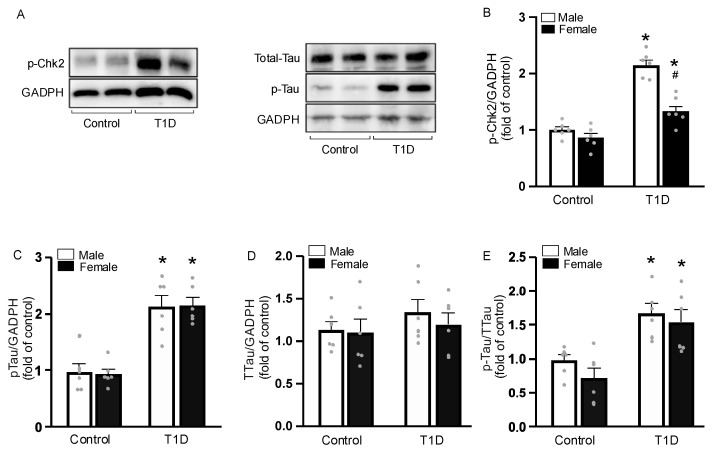
T1D leads to an increase in Chk2 and Tau phosphorylation in CASMCs. Representative image of Chk2 and Tau (**A**). Ratio between Chk2 and GADPH in CASMCs (**B**). Ratio between p-Tau and GADPH (**C**); total Tau and GADPH (**D**); and between p-Tau and total Tau (**E**) in CASMCs. Each dot represents one mouse. Analyzed by two-way ANOVA. * *p* < 0.05 compared with the control; ^#^ *p* < 0.05 compared with males. Data are presented as the mean with a 95% CI (n = 6–8/group). T1D = Type 1 diabetes. CASMC = cerebral artery smooth muscle cell.

T1D pathogenesis and progression is influenced by oxidative stress brought on by excessive free radicals, which can then result in genomic damage, particularly DNA damage. Therefore, we evaluated Chk2 phosphorylation as a means of assessing the extent of DNA damage. Representative images of Chk2 Western blotting are shown in Figure 6A. T1D mice had a significant increase in *p*-Chk2, but males had more significant DNA damage (Figure 6B).

### 3.7. Sex-Specific Tau Phosphorylation

Improper glucose metabolism due to nutrient deficiencies in the membrane of neurons can lead to neurodegeneration, leading to oxidative stress in mitochondria and cell death. Mitochondrial bioenergetic dysfunction and increased mitochondrial ROS production causes neuronal atrophy and eventual cell death in Alzheimer’s disease and other neuropathological conditions. Human tauopathies, such as Alzheimer’s disease, and mitochondrial dysfunction have both been linked to tau protein. We assessed this protein’s expression in CASMCs by Western blotting. As expected, we demonstrated that diabetes raises tau phosphorylation. In this work, we demonstrate that both males and females with T1D have a large increase in tau phosphorylation levels (Figure 6C–E).

## 4. Discussion

Our current study sought to elucidate the role of diabetes in the development of VCID using a STZ-induced T1D mouse model. Herein, we also investigated sex differences in in T1D-induced VCID. Moreover, we further explored the potential signaling molecules in male and female T1D. It is known that female mice have greater resistance to STZ-induced T1D compared to males, perhaps owing to the protective effects of estrogen on pancreatic islet cells [26,27]. Estrogen levels may control blood glucose levels [28].

**Figure 7:**
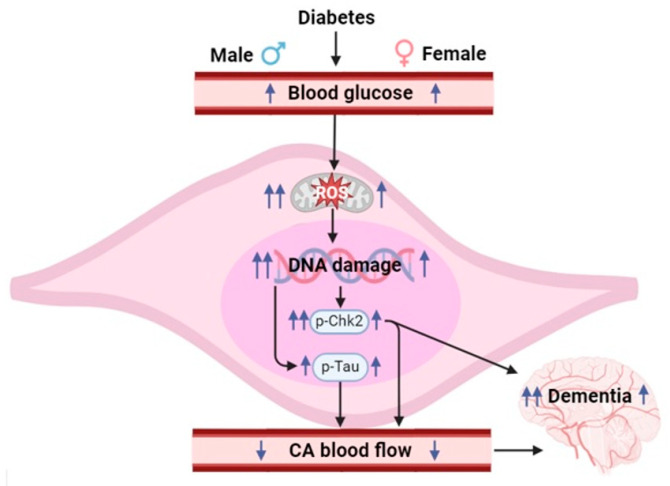
T1D causes increased glucose levels in blood, increased mitochondrial ROS production in CASMCs, DNA damage, increased Chk2 and Tau phosphorylation, and decreased cerebral blood flow, thereby leading to cognitive impairment, memory loss, and VCID. Moreover, mitochondrial complex III-derived ROS, DNA damage, and VCID are stronger in male than female T1D mice.

There is a strong correlation between high blood glucose levels and cognitive impairment (Figure 7). Higher glucose levels are known to be associated with an increased risk of dementia [29]. Clinical studies have shown a lower incidence of vascular dementia in women compared with men, particularly at younger ages [30]. T1D individuals exhibit significant cognitive impairments in visual attention, mental flexibility, and perception [31].

In the current study, we employed the open field, nest building, object position, and novel object recognition tests to assess cognitive and memory functions [32,33]. The open field test indicates that T1D mice of both sexes have a significant reduction time in the center of the box. As expected, T1D mice have shown a decrease in nest building score. We have also found that the novel object recognition, determined by the discrimination index, is decreased in T1D mice. Similarly, the novel object recognition, as measured by the discrimination index, is also largely diminished in T1D mice. All these findings imply a decline in both cognition and memory in T1D mice. Moreover, we have further found that more pronounced cognition and memory declines occur in T1D males than females.

Impairment of CBF leads to ischemia and subsequent neurocognitive decline and memory loss [34,35]. Diabetes predisposes patients to a wide range of perfusion defects, including VCID, due to its micro- and macro-vascular complications. We employed a laser speckle contrast imaging system to assess CBF in various brain regions, as reported previously [13]. The results unveil a marked reduction in CBF in the frontal, parietal, temporal, and pial zone anastomotic regions of T1D mice. This widespread reduction in CBF underscores the pervasive nature of vascular dysfunction in T1D and thus the cognitive impairment and memory loss in T1D mice. Our findings also emphasize the importance of maintaining adequate cerebral perfusion in T1D patients to mitigate the risk of VCID and other neurocognitive disorders [36].

Diabetes mellitus is closely associated with increased oxidative stress, a condition in which there is an imbalance between ROS production and antioxidant defense mechanisms. Chronic hyperglycemia activates several metabolic pathways that contribute to excessive ROS generation, such as the polyol pathway, the formation of advanced glycation products (AGEs), protein kinase C (PKC) activation, and mitochondrial dysfunction. These processes damage cells and tissues, promoting the development of typical diabetic vascular complications.

Several genes are involved in these mechanisms. Genes such as NOX1, NOX2 (CYBB), and NOX4, which encode subunits of the NADPH oxidase enzyme, are directly related to ROS production. Therefore, antioxidant genes like SOD2, GPX1, CAT, and GSR act to neutralize these reactive species. Polymorphisms in AKR1B1 (aldose reductase) are associated with enhanced activation of the polyol pathway. Additionally, the AGER gene, which encodes the RAGE receptor, mediates the harmful effects of AGEs by activating inflammatory and oxidative responses. Inflammatory genes such as TNF and IL6 also contribute to this oxidative environment. Thus, oxidative stress in diabetes results from complex interactions between hyperglycemia, metabolic pathways, and genetic factors [37,38].

In general, oxidative stress can refer to increased intracellular ROS production; complex III is a major site of ROS production [39]. Our findings establish that T1D causes increased ROS generation in CASMCs, with higher ROS production in isolated mitochondrial complex III from male T1D mice. Consistent with our data, complex III significantly contributes to ROS production in response to high glucose levels [40]. All these results show that sex-specific differences in ROS production at mitochondrial complex III may contribute to cognitive impairment, memory loss, and other behavioral alterations in T1D individuals.

The molecular mechanisms underlying T1D-related cerebrovascular outcomes involve multiple intracellular factors, including increased ROS generation. Indeed, T1D is characterized by significant loss of insulin-producing beta cells due to ROS-mediated inflammatory cytokine-induced apoptosis, and other cell death damage because of increased ROS and oxidative stress [41,42]. Diabetes increases ROS production and oxidative stress, leading to cell damage and even death [43]. We have observed the increased ROS production and oxidative stress in CASMCs from T1D mice. Estrogens modulate glucose metabolism and exert antioxidant properties, potentially contributing to the observed sex differences in oxidative stress and cognitive dysfunctions in T1D [44]. Our findings have for the first time established that T1D causes increased ROS generation in CASMCs, mitochondria, and complex III. This increased ROS production is higher in complex III in male than female T1D mice. Thus, there are lower levels of cerebrovascular oxidative stress in females compared to males, attributed to the presence of different gonadal hormones [36].

Our findings also uncover that the increased ROS production increases oxidative stress, contributing to DNA damage in T1D. The pathophysiology and progression of T1D are influenced by oxidative stress, resulting in DNA double-strand breaks [45]. We have further demonstrated the increased Chk2 expression in T1D, perhaps primarily establishing a strong correlation between DNA damage and increased ROS production [23]. Importantly, we have found that T1D-evoked DNA damage was more pronounced in male T1D mice compared to female T1D mice. This sex-specific difference in DNA damage may contribute to the more severe cognitive impairment and memory loss observed in male T1D mice. Our results further highlight the critical role of ROS-mediated DNA damage in the pathogenesis of T1D-induced VCID and suggest that targeting DNA repair mechanisms may be a promising therapeutic approach.

Tau hyperphosphorylation occurs in the cerebral cortex and hippocampus of diabetic mice, with no differences between sexes [24]. Diabetes-related cognitive impairment is a primary cause of dementia. The observed deficits in spatial and episodic memory tasks, particularly in male T1D mice, suggest a potential link between increased ROS production and tau protein dysregulation, a key pathological hallmark of neurodegenerative diseases like Alzheimer’s disease [46,47]. This finding suggests that while tau hyperphosphorylation is a crucial factor in T1D-induced cognitive impairment and memory loss, it may not be the primary driver of the observed sex differences in VCID severity. The increased tau phosphorylation in T1D mice may result from the combined effects of hyperglycemia, oxidative stress, and impaired insulin signaling [24]. Further investigation into the mechanisms underlying tau hyperphosphorylation in T1D may reveal novel therapeutic targets for preventing or mitigating cognitive decline and memory loss in diabetic patients.

To better understand the molecular mechanisms underlying VCID in T1D, we have made a linear regression analysis of blood glucose levels with ROS production and behavioral test results. Elevated blood glucose levels are highly correlated with dysfunctional behavioral tasks, particularly in male T1D mice, exhibiting lower scores in open field and novel object recognition tests. This establishes that major aspects of metabolic diseases and glucose metabolism vary significantly across sexes, supported by recent studies demonstrating sex variations in brain metabolism [48,49].

T1D mice have shown more cognitive deficits in various behavior tests, aligning with the neuroprotective effects of estrogenic compounds in the cerebral cortex [50]. We have observed significant oxidative stress, as indicated by the increased O_2_^−^, H_2_O_2_, and other ROS, in both male and female T1D CASMCs. This oxidative stress is higher in male than female T1D mice. Noticeably, these novel findings described herein are of great significance to better understand our current knowledge of diabetic VCID. Estrogens modulate glucose metabolism and exert antioxidant properties, potentially contributing to the observed sex differences in oxidative stress and associated cognitive function in T1D [44]. Our findings also suggest that sex-specific differences in ROS production at mitochondrial complex III may contribute to cognitive impairments and behavioral alterations in T1D individuals, particularly in sustained attention, mental flexibility, and episodic memory processing.

The potential limitations of this study should be acknowledged. Behavioral assessments such as the open field, nest building, object position, and novel object recognition tests, while widely used, may have limited sensitivity in small groups to detect subtle cognitive or memory changes, particularly at early time points. Although sex differences were identified, the underlying hormonal and molecular contributors were not directly manipulated or controlled, limiting causal interpretation. While correlations between ROS production, DNA damage, tau phosphorylation, and behavioral impairments were observed, these associations do not establish direct mechanistic causality, and further interventional studies are warranted. Finally, using the STZ-induced T1D model may not entirely mimic human T1D.

In conclusion, our study reveals for the first time that T1D causes the increased ROS production in mouse CASMCs, mitochondria, and complex III in male and female mice. The increased ROS production results in extensive DNA damage (i.e., Chk2 phosphorylation) and tau phosphorylation; these two responses lead to severe cognitive impairment, memory loss, and VCID. Moreover, greater ROS production at complex III, greater DNA damage, and more severe VCID occur in male then female T1D mice. These novel findings evidently highlight not only the importance of mitochondrial complex III ROS-dependent DNA damage and tau phosphorylation signaling in mediating T1D-induced VCID, but also the critical role of greater ROS-relied DNA damage in more severe VCID in male than female T1D mice.

## Data Availability

The original contributions presented in this study are included in the article. Further inquiries can be directed to the corresponding author(s).

## References

[1] Magliano D.J., Boyko E.J. IDF Diabetes Atlas. https://diabetesatlas.org/resources/idf-diabetes-atlas-2025/.

[2] Parker E.D., Lin J., Mahoney T., Ume N., Yang G., Gabbay R.A., ElSayed N.A., Bannuru R.R. (2024). Economic Costs of Diabetes in the U.S. in 2022. Diabetes Care.

[3] Syed F.Z. (2022). Type 1 Diabetes Mellitus. Ann. Intern. Med..

[4] Mooradian A.D. (1988). Diabetic complications of the central nervous system. Endocr. Rev..

[5] Santos E.W., Khatoon S., Di Mise A., Zheng Y.M., Wang Y.X. (2023). Mitochondrial Dynamics in Pulmonary Hypertension. Biomedicines.

[6] Truong L.N., Santos E.W., Zheng Y.M., Wang Y.X. (2023). Rieske Iron-Sulfur Protein Mediates Pulmonary Hypertension Following Nicotine/Hypoxia Co-Exposure. Am. J. Respir. Cell Mol. Biol..

[7] Busija D.W., Rutkai I., Dutta S., Katakam P.V. (2016). Role of Mitochondria in Cerebral Vascular Function: Energy Production, Cellular Protection, and Regulation of Vascular Tone. Compr. Physiol..

[8] Kautzky-Willer A., Harreiter J., Pacini G. (2016). Sex and Gender Differences in Risk, Pathophysiology and Complications of Type 2 Diabetes Mellitus. Endocr. Rev..

[9] Jiang Q., Xu H., Yan J., Xu Q., Zheng Y., Li C., Zhao L., Gao H., Zheng H. (2020). Sex-specific metabolic alterations in the type 1 diabetic brain of mice revealed by an integrated method of metabolomics and mixed-model. Comput. Struct. Biotechnol. J..

[10] Stenberg L., Dahlin L.B. (2014). Gender differences in nerve regeneration after sciatic nerve injury and repair in healthy and in type 2 diabetic Goto-Kakizaki rats. BMC Neurosci..

[11] Zeinivand M., Nahavandi A., Zare M. (2020). Deferoxamine regulates neuroinflammation and oxidative stress in rats with diabetes-induced cognitive dysfunction. Inflammopharmacology.

[12] Dong L., Zheng Y.M., Van Riper D., Rathore R., Liu Q.H., Singer H.A., Wang Y.X. (2008). Functional and molecular evidence for impairment of calcium-activated potassium channels in type-1 diabetic cerebral artery smooth muscle cells. J. Cereb. Blood. Flow. Metab..

[13] Salinero A.E., Robison L.S., Gannon O.J., Riccio D., Mansour F., Abi-Ghanem C., Zuloaga K.L. (2020). Sex-specific effects of high-fat diet on cognitive impairment in a mouse model of VCID. FASEB J..

[14] Denninger J.K., Smith B.M., Kirby E.D. (2018). Novel Object Recognition and Object Location Behavioral Testing in Mice on a Budget. J. Vis. Exp..

[15] Lueptow L.M. (2017). Novel Object Recognition Test for the Investigation of Learning and Memory in Mice. J. Vis. Exp..

[16] Deacon R.M. (2006). Assessing nest building in mice. Nat. Protoc..

[17] Clayton D.A., Shadel G.S. (2014). Isolation of mitochondria from tissue culture cells. Cold Spring Harb. Protoc..

[18] Wang Q.S., Zheng Y.M., Dong L., Ho Y.S., Guo Z., Wang Y.X. (2007). Role of mitochondrial reactive oxygen species in hypoxia-dependent increase in intracellular calcium in pulmonary artery myocytes. Free Radic. Biol. Med..

[19] Yang Z., Song T., Truong L., Reyes-García J., Wang L., Zheng Y.M., Wang Y.X. (2020). Important Role of Sarcoplasmic Reticulum Ca. Antioxid. Redox Signal..

[20] Zheng Y.M., Wang Q.S., Liu Q.H., Rathore R., Yadav V., Wang Y.X. (2008). Heterogeneous gene expression and functional activity of ryanodine receptors in resistance and conduit pulmonary as well as mesenteric artery smooth muscle cells. J. Vasc. Res..

[21] Korde A.S., Yadav V.R., Zheng Y.M., Wang Y.X. (2011). Primary role of mitochondrial Rieske iron-sulfur protein in hypoxic ROS production in pulmonary artery myocytes. Free Radic. Biol. Med..

[22] Panov A., Dikalov S., Shalbuyeva N., Taylor G., Sherer T., Greenamyre J.T. (2005). Rotenone model of Parkinson disease: Multiple brain mitochondria dysfunctions after short term systemic rotenone intoxication. J. Biol. Chem..

[23] van Jaarsveld M.T.M., Deng D., Ordoñez-Rueda D., Paulsen M., Wiemer E.A.C., Zi Z. (2020). Cell-type-specific role of CHK2 in mediating DNA damage-induced G2 cell cycle arrest. Oncogenesis.

[24] Clodfelder-Miller B.J., Zmijewska A.A., Johnson G.V., Jope R.S. (2006). Tau is hyperphosphorylated at multiple sites in mouse brain in vivo after streptozotocin-induced insulin deficiency. Diabetes.

[25] Zuloaga K.L., Johnson L.A., Roese N.E., Marzulla T., Zhang W., Nie X., Alkayed F.N., Hong C., Grafe M.R., Pike M.M. (2016). High fat diet-induced diabetes in mice exacerbates cognitive deficit due to chronic hypoperfusion. J. Cereb. Blood Flow Metab..

[26] Kim B., Kim Y.Y., Nguyen P.T.T., Nam H., Suh J.G. (2020). Sex differences in glucose metabolism of streptozotocin-induced diabetes inbred mice (C57BL/6J). Appl. Biol. Chem..

[27] Furman B.L. (2021). Streptozotocin-Induced Diabetic Models in Mice and Rats. Curr. Protoc..

[28] Le May C., Chu K., Hu M., Ortega C.S., Simpson E.R., Korach K.S., Tsai M.J., Mauvais-Jarvis F. (2006). Estrogens protect pancreatic beta-cells from apoptosis and prevent insulin-deficient diabetes mellitus in mice. Proc. Natl. Acad. Sci. USA.

[29] Crane P.K., Walker R., Hubbard R.A., Li G., Nathan D.M., Zheng H., Haneuse S., Craft S., Montine T.J., Kahn S.E. (2013). Glucose levels and risk of dementia. N. Engl. J. Med..

[30] Akhter F., Persaud A., Zaokari Y., Zhao Z., Zhu D. (2021). Vascular Dementia and Underlying Sex Differences. Front. Aging. Neurosci..

[31] Kim H.G. (2019). Cognitive dysfunctions in individuals with diabetes mellitus. Yeungnam Univ. J. Med..

[32] Keleher M.R., Zaidi R., Patel K., Ahmed A., Bettler C., Pavlatos C., Shah S., Cheverud J.M. (2018). The effect of dietary fat on behavior in mice. J. Diabetes Metab. Disord..

[33] Kraeuter A.K., Guest P.C., Sarnyai Z. (2019). The Nest Building Test in Mice for Assessment of General Well-Being. Methods Mol. Biol..

[34] Kamada H., Yu F., Nito C., Chan P.H. (2007). Influence of hyperglycemia on oxidative stress and matrix metalloproteinase-9 activation after focal cerebral ischemia/reperfusion in rats: Relation to blood-brain barrier dysfunction. Stroke.

[35] Liu C., Wu J., Zou M.H. (2012). Activation of AMP-activated protein kinase alleviates high-glucose-induced dysfunction of brain microvascular endothelial cell tight-junction dynamics. Free Radic. Biol. Med..

[36] Gannon O.J., Robison L.S., Custozzo A.J., Zuloaga K.L. (2019). Sex differences in risk factors for vascular contributions to cognitive impairment & dementia. Neurochem. Int..

[37] Azarova I., Polonikov A., Klyosova E. (2023). Molecular Genetics of Abnormal Redox Homeostasis in Type 2 Diabetes Mellitus. Int. J. Mol. Sci..

[38] Pickering R.J., Rosado C.J., Sharma A., Buksh S., Tate M., de Haan J.B. (2018). Recent novel approaches to limit oxidative stress and inflammation in diabetic complications. Clin. Transl. Immunol..

[39] Forkink M., Basit F., Teixeira J., Swarts H.G., Koopman W.J.H., Willems P.H.G.M. (2015). Complex I and complex III inhibition specifically increase cytosolic hydrogen peroxide levels without inducing oxidative stress in HEK293 cells. Redox Biol..

[40] Forrester S.J., Kikuchi D.S., Hernandes M.S., Xu Q., Griendling K.K. (2018). Reactive Oxygen Species in Metabolic and Inflammatory Signaling. Circ. Res..

[41] Berchtold L.A., Prause M., Størling J., Mandrup-Poulsen T. (2016). Cytokines and Pancreatic β-Cell Apoptosis. Adv. Clin. Chem..

[42] Wolff S.P. (1993). Diabetes mellitus and free radicals. Free radicals, transition metals and oxidative stress in the aetiology of diabetes mellitus and complications. Br. Med. Bull..

[43] Ahmad W., Ijaz B., Shabbiri K., Ahmed F., Rehman S. (2017). Oxidative toxicity in diabetes and Alzheimer’s disease: Mechanisms behind ROS/RNS generation. J. Biomed. Sci..

[44] Rettberg J.R., Yao J., Brinton R.D. (2014). Estrogen: A master regulator of bioenergetic systems in the brain and body. Front. Neuroendocrinol..

[45] Giovannini C., Piaggi S., Federico G., Scarpato R. (2014). High levels of γ-H2AX foci and cell membrane oxidation in adolescents with type 1 diabetes. Mutat. Res..

[46] Madhusudhanan J., Suresh G., Devanathan V. (2020). Neurodegeneration in type 2 diabetes: Alzheimer’s as a case study. Brain. Behav..

[47] Guo L., Tian J., Du H. (2017). Mitochondrial Dysfunction and Synaptic Transmission Failure in Alzheimer’s Disease. J. Alzheimer’s Dis..

[48] Wu C.Y., Lin Y.H., Hsieh H.H., Lin J.J., Peng S.L. (2021). Sex Differences in the Effect of Diabetes on Cerebral Glucose Metabolism. Biomedicines.

[49] Tiano J.P., Delghingaro-Augusto V., Le May C., Liu S., Kaw M.K., Khuder S.S., Latour M.G., Bhatt S.A., Korach K.S., Najjar S.M. (2011). Estrogen receptor activation reduces lipid synthesis in pancreatic islets and prevents β cell failure in rodent models of type 2 diabetes. J. Clin. Investig..

[50] Crespo-Castrillo A., Yanguas-Casás N., Arevalo M.A., Azcoitia I., Barreto G.E., Garcia-Segura L.M. (2018). The Synthetic Steroid Tibolone Decreases Reactive Gliosis and Neuronal Death in the Cerebral Cortex of Female Mice After a Stab Wound Injury. Mol. Neurobiol..

